# Prevalence of Hepatitis C Virus Infection and Its Risk Factors among Patients Attending Rwanda Military Hospital, Rwanda

**DOI:** 10.1155/2017/5841272

**Published:** 2017-01-26

**Authors:** Esperance Umumararungu, Fabien Ntaganda, John Kagira, Naomi Maina

**Affiliations:** ^1^Department of Molecular Biology and Biotechnology, Institute of Basic Sciences, Technology and Innovation (PAUISTI), Pan African University, P.O. Box 6200-00200, Nairobi, Kenya; ^2^Rwanda Military Hospital, P.O. Box 3377, Kanombe, Kigali, Rwanda; ^3^Department of Animal Sciences, Faculty of Agriculture, Jomo Kenyatta University of Agriculture and Technology (JKUAT), Nairobi, Kenya; ^4^Department of Biochemistry, School of Biomedical Sciences, Jomo Kenyatta University of Agriculture and Technology (JKUAT), P.O. Box 62000-00200, Nairobi, Kenya

## Abstract

In Rwanda, the prevalence of viral hepatitis (HCV) is poorly understood. The current study investigated the prevalence and risk factors of HCV infection in Rwanda. A total of 324 patients attending Rwanda Military Hospital were randomly selected and a questionnaire was administered to determine the risk factors. Blood was collected and screened for anti-HCV antibodies and seropositive samples were subjected to polymerase chain reaction method. Hematology abnormalities in the HCV infected patients were also investigated. Anti-HCV antibody and active HCV infection were found in 16.0% and 9.6% of total participants, respectively. Prevalence was highest (28.4%; 19/67) among participants above 55 years and least (2.4%; 3/123) among younger participants (18–35 years). There was a significant (*P* = 0.031) relationship between place of residence and HCV infection with residents of Southern Province having significantly higher prevalence. The hematological abnormalities observed in the HCV infected patients included leukopenia (48.4%; 15/52), neutropenia (6.5%; 2/52), and thrombocytopenia (25.8%; 8/52). The HCV infection was significantly higher in the older population (>55 years) and exposure to injection from traditional practitioners was identified as a significant (*P* = 0.036) risk factor of infection. Further studies to determine the factors causing the high prevalence of HCV in Rwanda are recommended.

## 1. Introduction

Globally, about 130–150 million people are living with chronic HCV infection [1] with about 350,000–500,000 lives lost every year [2, 3]. Infection begins as acute and usually asymptomatic during early stages [4, 5]. In most untreated cases, the infection progresses into chronic infections and gradually develops liver fibrosis which then leads to cirrhosis, liver damage, and hepatocellular carcinoma (HCC) [6]. In Africa, the prevalence of HCV is between 0.1% and 17.5%. In Rwanda, prevalence of HCV infection has been reported in specific groups of the populations such as in pregnant women and in patients infected with tuberculosis and HIV [7–10]. However, the prevalence of HCV for the general population is not clear. A prevalence of 4.9% was estimated in a 2011 study [11] but a recent review by Karoney and Siiki indicates that this figure could be an underestimation [12]. This is because of challenges such as barriers to screening, cost-related factors, and inadequate knowledge and awareness of hepatitis C [13].

There are many risk factors of acquiring HCV infection. In the Sub-Saharan Africa, practices such as dental surgery, therapeutic injection, intravenous drug, and age have been reported as major risk factors associated with HCV infection [14–16]. In Rwanda, the exact risk factors of HCV infection are not clear.

The aims of the current study were to determine the prevalence, hematological abnormalities, and risk factors associated with HCV infection in patients attending Rwanda Military Hospital (RMH), a national referral hospital in Rwanda.

## 2. Materials and Method

### 2.1. Study Site, Study Design, and Population

The study was conducted at the Rwanda Military Hospital (RMH), a national referral and teaching hospital with a bed capacity of 500 located at Kanombe, in Kicukiro District of Kigali Province, Rwanda. The hospital provides health care services to around 40,000 to 50,000 patients including military personnel every year. The study utilized out-patients referred for laboratory examination and who were 18 years and above. Using Kish Leslie formula [17] for cross-sectional studies and an average prevalence of 3.5% from two previous studies [7, 10], a sample size of 324 was determined. The sample size was spread across a two-month period and subsequently divided into 9 patients per day per month. Therefore, 9 patients/day attending laboratory were randomly enrolled after they had given informed consent. Information about the study was given in English, French, or Kinyarwanda. A structured questionnaire to obtain bio-data and exposure risks such as blood transfusion, organ transplantation, living with HCV infected patients, hospital admission, previous surgery, and hospitalization; accidental needle stick injuries treated by traditional doctor and travelling outside were also administered.

### 2.2. Blood Collection, Processing, and HCV Antibody Screening

About 10 mL of venous blood from the median cubital vein was collected into ethylenediaminetetraacetic acid (EDTA) tubes. Five milliliters of this blood was centrifuged for 10 min at 3000 RPM to obtain plasma. An aliquot of 500 *μ*L of each plasma sample was made and screened for anti-HCV antibody using Cypress anti-HCV dipstick following instructions of the manufacturer's manual [18]. The sensitivity and specificity of the assay used are 95.29% and 98.75%, respectively. The remaining plasma samples of all anti-HCV antibody positive samples were aliquoted in two cryovials and stored at −80°C for HCV RNA testing.

### 2.3. Hematological Analysis

EDTA blood samples of seropositive participants (5 mL each) were subjected to hematology analysis using Sysmex XS800i Automatic Analyzer [19]. Full blood count including white blood cell differential counts was determined. The erythrocyte sedimentation rate (ESR) was also measured using Westergren method [20].

### 2.4. HCV RNA Testing

Active RNA infection of all seropositive samples was confirmed by HCV RNA testing. One vial of cryopreserved plasma was retrieved from −80°C freezer and thawed at room temperature for 15 min. Following manufacturer's manual, HCV RNA was automatically extracted from 650 *μ*L plasma samples, reverse transcribed into complementary DNA (cDNA), and amplified and amplicons were detected using Cobas AmpliPrep/Cobas TaqMan HCV machine, version 2 [21]. Detection of viremia was recorded as target detected or target not detected.

### 2.5. Statistical Analysis

All data were entered and analyzed in Statistical Package for the Social Sciences (SPSS) software version 20.0 for windows [22]. Comparison between categorical variables was computed using Fisher's exact test or Pearson Chi-square test. A *p* value of less than 0.05 was considered statistically significant.

### 2.6. Ethical Statement

This study was approved by the Institutional Ethical Review Committee (IRC) of the Rwanda Military Hospital.

## 3. Results

**Figure 1:**
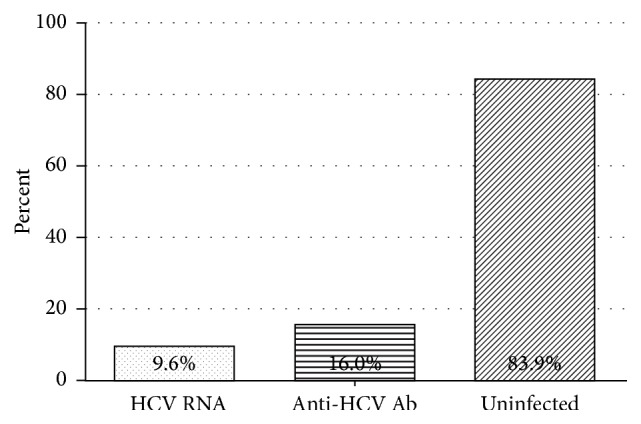
Prevalence of HCV Infection among patients attending laboratory at RMH.

Of the 324 study participants, 133 (41.0%) were males and 191 (59.0%) were females. The mean age of the participants was 42.32 years. The demographic characteristics of the study participants are shown in Table 1. Serological testing for HCV of all 324 participants revealed that 16.0% (52/324) of them were seropositive. Confirmation of active HCV RNA infection among seropositive participants revealed that, of the 52 anti-HCV positive participants, 31 (59.6%) had detectable viremia. Thus, an overall active HCV prevalence of 9.6% (31/52) was recorded (Figure 1).

Prevalence of active HCV infection was higher (9.8%, 3/31) in males than in females (9.4%, 18/31). However, this was not significant (*p* = 0.916, *χ*^2^ = 0.011) (Table 2).

The participants were divided into three age categories. Anti-HCV antibodies were detected in 41.8% of participants older than 55 years, 11.9% middle age participants (36–55 years), and 6.5% younger participants (18–35 years). In general, it was observed that prevalence of active HCV infection increased significantly with increasing age (*p* = 0.001) (Table 3).

**Figure 2:**
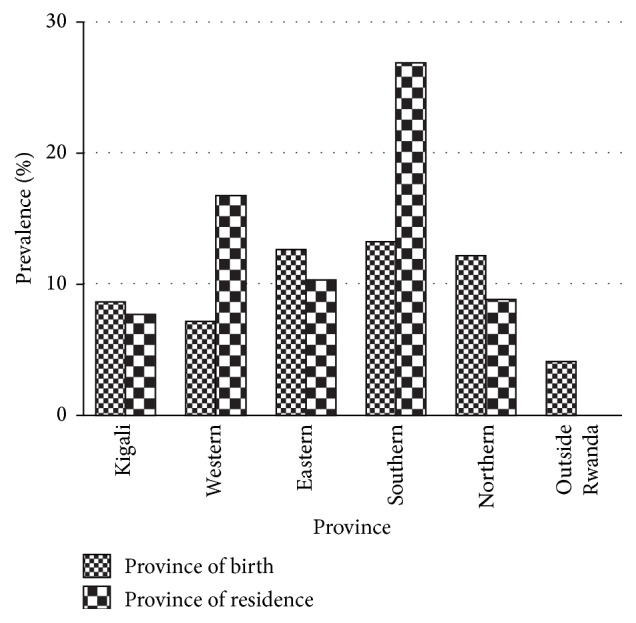
Prevalence of HCV RNA infection according to birth place and residence.

Prevalence of active HCV infection was highest among participants born in Southern Rwanda (13.1%, 13/86) and least among participants born outside Rwanda (3.9%, 3/74). However no significant relationship was found between participant's birth place and active HCV RNA infection (*p* = 0.390, *χ*^2^ = 5.213). In contrast, there was a significant (*p* = 0.031; *χ*^2^ = 10.627) relationship between participants place of residence and active RNA infection. Infection was highest among residents of Southern followed by Western, Eastern, Northern, and Kigali (Figure 2).

HCV infection was most commonly found among widows/widowers and none of divorced participants was positive as shown in Table 4. However, no significant relationship was found (*p* > 0.05)

Comparison of risk factors of acquiring HCV infection among participants was also analyzed (Table 5). It was observed that participants who had received injection from traditional practitioners had a significant (*p* = 0.036) chance of having HCV infection. Similarly, persons who had lost a relative through hepatitis C (HC) and those with no educational background were more likely to have HCV infection.

Hematological abnormalities observed among the HCV infected participants were leukocytopenia (48.4%), lymphopenia (3.2%), neutropenia (6.5%), and thrombocytopenia (25.8%) (Table 6). The prevalence of neutrophil abnormalities (neutropenia) was significantly (*p* = 0.033) higher in males than in females. Leukocytopenia was more prevalent but not significant (*p* > 0.05) in males (53.8%) than in females (44.4%). In contrast, thrombocytopenia was more common in females (33.3%) than males (15.4%). Only one female (5.6%) had anemia. Erythrocytes Sedimentation Rate (ESR) was recorded under two categories; >20 mm/hr and <20 mm/hr. Overall 5 (16.1%) HCV patients had ESR values above 20 mm/hr, with 23.1% and 11.1% being males and females, respectively.

## 4. Discussion

Viral hepatitis is a major infectious disease of global concern [23]. In Sub-Saharan Africa, viral hepatitis due to HCV infection is highly prevalent but the extent of the disease burden may be underreported [12, 24].

Prevalence of HCV infection varies across different regions and populations [25]. In Africa, prevalence of HCV infection reported so far has focused on a specific group of the population mostly relying on the error-prone antibody testing method [9, 10, 26, 27]. In this study, participants were first screened against the presence of HCV antibodies using rapid diagnostic strips. Seropositive cases were then subjected to the more sensitive polymerase chain reaction method. A high seroprevalence of 16.0% was reported in patients attending the national referral hospital in Rwanda. This figure is comparatively higher than the 1.3% seroprevalence reported by Kateera et al. with the same Cypress anti-HCV dipstick [7]. This test has 98.7% relative specificity for antibodies against both structural and nonstructural proteins of HCV [15]. However, the assay generally lacks the discriminatory ability to differentiate between antibodies resulting from active infection and previously cleared infection [28]. It is recommended that all patients having anti-HCV antibodies are subjected to further confirmatory testing thereby eliminating such false positives [29]. In this study, the overall prevalence of active HCV infection was 9.6% (31/324). This prevalence indicates the cost-effectiveness of screening using serological tests and confirmation by RNA. The current cost of RNA testing in Rwanda is about $100.00, limiting the general public to only HCV antibody screening.

This study noted infection was higher in males than in females. Many studies have reported similar findings [30]. This high infection rate in males than in females may be due to spontaneous clearance of acute infection in females [31]. The reason to this clearance has been attributed occurrence of certain genetic factors such as IL28B genetic variants in females [5, 32]. Indeed even the hematological abnormalities noted in this study were more in males than females; however there is a significant association of HCV infection and thrombocytopenia; this association has been reported earlier [33]. Similarly, prevalence of active HCV infection increased significantly with age and this has been reported in Uganda [34] and in Madagascar [16] and this may be due to frequent exposure [35]. With the high prevalence being reported in patients older than 55 years, more screening for HCV can focus on this age group.

In developing countries various risk factors associated with acquiring HCV infection have been reported in various studies. Identification of risk factor enables appropriate control strategies to be developed. In this study hospital based risk factors were not found to be significant. This is in contrast to a study in Ethiopia where history of hospitalization, tooth extraction, and blood transfusion were identified as major risk factors HCV infection [27].

The current study showed that HCV prevalence was highest in widows/widower and also in married individuals. This high prevalence may imply that transmission via sex is important in Rwanda. In order to break the cycle of HCV transmission, it is highly recommended that, screening among sexually active individuals be performed.

A significant observation made from this study is that HCV prevalence was highest among persons residing in Southern Rwanda followed by Western Rwanda. The high prevalence of infection in this part of Rwanda may be due to migration of persons at the border with Burundi where prevalence is highest in East Africa [12]. It is therefore recommended that more screening for persons living in Southern Rwanda and at various ports of entry be done.

In the present study, alanine aminotransferase (ALT) and aspartate transaminase (AST) values were not measured. Even though their values may not play a role in diagnosis of HCV, they may be important in disease management [35, 36]. They were however not tested due to the limitation of the study design. Liver biopsy was also not performed in this study due to obvious reasons such as cost, risk of complications, and need for additional health care resources [37]. The study was also unable to explore the issues relating to prevalence of HIV and HBV coinfections with HCV as these two infections have been shown to impact negatively the clinical course of chronic HCV infection [38–40]. With the high prevalence (9.6%) of active HCV infection reported, a study the HIV and HBV coinfections with HCV would be of high priority.

## 5. Conclusion

The current study shows a high prevalence of HCV infection in Rwanda. Infection was more likely to occur in older persons than younger ones. Unsafe injections by traditional practitioners were of significant exposure risk of contracting HCV infection. The results further indicate that a person's place of birth and residence could determine their HCV status. Although, hematological abnormalities observed in this study were widespread among infected participants. This study indicates the need for a larger study to ascertain extent of HCV infections in Rwanda.

## Figures and Tables

**Table 1:** Demographic characteristics of participants in the study.

Variable	Number of Participants (%)
Gender	
Male	133 (41.0%)
Female	191 (59.0%)
Age group (yrs)	
18–35	123 (38.0%)
36–55	143 (41.4%)
>55	67 (20.7%)
Marital Status	
Single	73 (22.5%)
Married	207 (63.9%)
Divorced	37 (11.4%)
Widow	7 (2.2%)
Employment sector	
Commerce	71 (21.9%)
Health	13 (4.0%)
Agriculture (farming)	46 (14.2%)
Security	35 (10.8%)
Transport	8 (2.5%)
Education and religion	44 (13.6%)
Technical staff	15 (4.6%)
Unemployed	73 (22.5%)
Other	19 (5.9%)
Place of birth	
Kigali	47 (14.5%)
Southern	103 (31.8%)
Northern	33 (10.2%)
Eastern	39 (12.0%)
Western	24 (7.4%)
Abroad	78 (24.1%)
Place of Residence	
Kigali	241 (74.4%)
Southern	27 (8.3%)
Northern	22 (6.8%)
Eastern	27 (8.3%)
Western	7 (2.2%)
Level of Education	
Primary	118 (36.4%)
Secondary	119 (36.7%)
Tertiary	73 (22.5%)
None	14 (4.3%)

**Table 2:** Gender wise distribution of anti-HCV antibodies and HCV RNA.

Gender	Number of participants	Anti-HCV +ve	HCV RNA +ve
Male	133	24 (18.0%)	13 (9.8%)
Female	191	28 (14.7%)	18 (9.4%)

*Total*	*324*	*52 (16.0%)*	*31 (9.6%)*

**Table 3:** Age wise Prevalence of anti-HCV antibodies and HCV RNA among study participants.

Age group (yrs)	Number of participants	Anti-HCV +ve	HCV RNA
18–35	123	8 (6.5%)	3 (2.4%)
36–55	134	16 (11.9%)	9 (6.7%)
>55	67	28 (41.8%)	19 (28.4%)

* Total*	*324*	*52 (16.0%)*	*31 (9.6%)*

**Table 4:** Distribution active HCV RNA infection according to marital status.

Marital Status	Number of Participants	HCV RNA
Single	73	6 (8.2%)
Married	207	18 (8.7%)
Divorced	7	0 (0.0%)
Widow/widower	37	7 (18.9%)

*Total*	*324*	*31 (9.6%)*

**Table 5:** Risk/exposure factors of HCV infection among 324 study participants.

Risk factor	*N*	HCV RNA +ve	*p* value
Blood transfusion			0.939
Yes	19	2 (10.5%)	
No	304	29 (9.5%)	
Relative died of HC			0.052
Yes	57	8 (14.0%)	
No	261	21 (8.0%)	
Unknown	6	2 (33.3%)	
Living with infected person			0.271
Yes	51	7 (13.7%)	
No	273	24 (8.8%)	
Hospitalized			0.354
Yes	151	12 (7.9%)	
No	173	19 (11.0%)	
Surgeries performed			0.717
Yes	71	6 (8.5%)	
No	253	25 (9.9%)	
Needle Injection by traditional practitioners			0.036^*∗*^
Yes	19	5 (26.3%)	
No	304	26 (8.3%)	
Treated by traditional doctor			0.195
Yes	93	12 (12.9%)	
No	231	19 (8.2%)	
Travelled outside before			0.197
Yes	195	22 (11.3%)	
No	129	9 (7.0%)	
Education background			0.097
Primary	118	15 (12.7%)	
Secondary	119	10 (8.4%)	
Tertiary	73	3 (4.1%)	
None	14	3 (21.4%)	

^*∗*^Significant at 95% confidence interval.

**Table 6:** Prevalence of haematological abnormalities among HCV infected participants.

Abnormality	*N* (%)	Gender		*p*-value	Age (yrs)			*p* value
		Male	Female		18–35	36–55	>55	
Leukopenia	15 (48.4%)	7 (53.8%)	8 (44.4%)	0.605	3 (75%)	2 (25%)	10 (52.6%)	0.221
Lymphopenia	1 (3.2%)	1 (7.7%)	0 (%)	0.403	0	0	1 (5.3%)	0.853
Neutropaenia	2 (6.5%)	1 (7.7%)	1 (5.6%)	0.332	1 (25%)	0	1 (5.3%)	0.296
Thrombocytopenia	8 (25.8%)	2 (15.4%)	6 (33.3%)	0.260	0	0	8 (42.1%)	0.033^*∗*^
Anaemia	1 (3.2%)	0 (0%)	1 (5.6%)	0.388	0	0	1 (5.3%)	0.722
ESR	5 (16.1%)	3 (23.1%)	2 (11.1%)	0.537	0	2 (25%)	3 (15.8%)	0.539

^*∗*^Significant at 95% confidence interval. Computed using Chi-square test. Reference range of hematological variables: total WBC count (4.50–11.50 [10^3^/*μ*L]) lymphocytes (Female: 1.30–3.70, males: 1.20–4.80 [10^3^/*µ*L]); neutrophils (Females: 1.10–4.40, males: 2.30–8.10 [10^3^/*µ*L]); platelets (150–540 [10^3^/*µ*L]), haemoglobin (Females: 11.0–17.0, Males: 12.0–18.0 [g/dL]), and ESR (<20 mm/hr).
